# Crimean-Congo haemorrhagic fever (CCHF) virus-specific antibody detection in blood donors, Castile-León, Spain, summer 2017 and 2018

**DOI:** 10.2807/1560-7917.ES.2020.25.10.1900507

**Published:** 2020-03-12

**Authors:** Lía Monsalve Arteaga, Juan Luis Muñoz Bellido, María Carmen Vieira Lista, María Belén Vicente Santiago, Pedro Fernández Soto, Isabel Bas, Nuria Leralta, Fernando de Ory Manchón, Ana Isabel Negredo, María Paz Sánchez Seco, Montserrat Alonso Sardón, Sonia Pérez González, Ana Jiménez del Bianco, Lydia Blanco Peris, Rufino Alamo-Sanz, Roger Hewson, Moncef Belhassen-García, Antonio Muro

**Affiliations:** 1 Infectious and Tropical Diseases Group (e-INTRO). IBSAL-CIETUS (Biomedical Research Institute of Salamanca-Research Center for Tropical Diseases at the University of Salamanca), Faculty of Pharmacy, University of Salamanca, Salamanca, Spain; 2 Instituto de Investigación Biomédica de Salamanca (IBSAL), Universidad de Salamanca, CSIC, Complejo Asistencial Universitario de Salamanca, Salamanca, Spain; 3 Servicio de Microbiología y Parasitología, Complejo Asistencial Universitario de Salamanca, Salamanca, Spain; 4 Departamento de Ciencias Biomédicas y del Diagnóstico, Universidad de Salamanca, Salamanca, Spain; 5 Arbovirus and Imported Viral Diseases Unit, Centro Nacional de Microbiología, Instituto de Salud Carlos III, Red de Investigación Colaborativa en Enfermedades Tropicales, Madrid, Spain; 6 Centro Nacional de Microbiología, Ciber en Salud Pública (CIBERESP), Instituto de Salud Carlos III, Majadahonda, Madrid, Spain; 7 Center for Hemodonation and Hemotherapy of Castilla y León (CHEMCYL), Valladolid, Spain; 8 Consejería de Sanidad Junta Castilla y León, Valladolid, Spain; 9 Public Health England, Porton Down, Wiltshire, Salisbury, United Kingdom

**Keywords:** Crimean-Congo haemorrhagic fever, CCHF, emerging diseases, re-emerging diseases, epidemiology, laboratory, tick-borne diseases, viral haemorrhagic fever, blood donor

## Abstract

**Background:**

Crimean-Congo haemorrhagic fever virus (CCHFV) is considered an emerging or even a probable re-emerging pathogen in southern Europe. Presence of this virus had been reported previously in Spain in 2010.

**Aim:**

We aimed to evaluate the potential circulation of CCHFV in western Spain with a serosurvey in asymptomatic adults (blood donors).

**Methods:**

During 2017 and 2018, we conducted a CCHFV serosurvey in randomly selected asymptomatic blood donors from western Spain. Three assays using specific IgG antibodies against CCHFV were performed: the VectoCrimea ELISA test, an in-house ELISA and indirect immunofluorescence (EuroImmun) test with glycoprotein and nucleoprotein.

**Results:**

A total of 516 blood donors participated in this cross-sectional study. The majority of the study participants were male (68.4%), and the mean age was 46.3 years. Most of the participants came from rural areas (86.8%) and 68.6% had contact with animals and 20.9% had animal husbandry practices. One in five participants (109/516, 21.1%) were engaged in at-risk professional activities such as agriculture and shepherding, slaughtering, hunting, veterinary and healthcare work (mainly nursing staff and laboratory technicians). A total of 15.3% of the participants were bitten by ticks in the days or months before the date of sampling. We detected anti-CCHFV IgG antibodies with two diagnostic assays in three of the 516 individuals and with one diagnostic assay in six of the 516 individuals.

**Conclusion:**

Seroprevalence of CCHFV was between 0.58% and 1.16% in Castile-León, Spain. This is the first study in western Spain that showed circulation of CCHFV in healthy people.

## Introduction

Crimean-Congo haemorrhagic fever (CCHF) is a tick-borne viral disease caused by the CCHF virus (CCHFV), an RNA negative single-stranded virus of the genus *Nairovirus* in the *Nairoviridae* family [1]. The virus has been identified in Africa, Asia and Europe in territories located south of the 50th North parallel, the area inhabited by the main vector, ticks of the genus *Hyalomma* spp. [2-5]. People living in rural areas, especially those involved in animal husbandry and slaughtering, are particularly at risk. Wild animals such as red deer and domestic animals including livestock can be reservoirs of the virus (they can become asymptomatically infected or host the infected hard-body ticks). Since cattle serve as habitual hosts, people can become infected by tick bites or by manipulating CCHFV-infected animals or their body fluids [6,7]. It is well known that CCHFV is implicated in outbreaks with a very high mortality rate (10–40%) [5,8]. This potential risk is the reason why CCHFV was included by the World Health Organization (WHO) as a priority pathogen for research and development [9-11].

CCHF is considered an emerging disease in southern Europe, with published reports from Albania, Bulgaria, Greece, Kosovo* and Turkey [12-19]. Moreover, imported cases have been detected in France and the United Kingdom (UK) in 2004 and 2013, respectively [20-22]. Filipe et al. reported the presence of IgG antibodies against CCHFV in asymptomatic individuals on the Iberian Peninsula in southern Portugal in 1984 [23]. Later, in 2010, CCHFV circulation was also identified for the first time in Spain, when the viral genome was detected in *Hyalomma* spp. ticks retrieved from wild red deer in Caceres (western Spain) 24]. In 2016, the first autochthonous human infection was recognised in a man who travelled to the province of Ávila, 300 km south-west of Caceres where the infected ticks had been identified [25]. After an extensive effort of public health vigilance, the virus was identified in ticks feeding on domestic and wild animals in western Spain [26-28]. This could be attributed to the silent circulation of CCHFV introduced some time ago from West Africa by migratory birds [26,29].

A study carried out in the area did not find antibodies against the virus in humans [30], however, this could have been due to small sample size. The aim of our present study was to evaluate the potential circulation of CCHFV in Castile-León by a serosurvey performed in asymptomatic adults. We decided to work with blood donors because this group resembles the healthy population of a given zone and we can infer the real situation in a specific area from a prevalence value in this group.

## Methods

### Study design

A descriptive, cross-sectional study was carried out between May 2017 and May 2018 in the Castile-León Hemotherapy and Hemodonation Centre in Valladolid, Spain, the centre that centralises blood product collection for the Autonomous Community of Castile-León with an area of 94,225 km^2^ and a total population of 2,409,164 inhabitants in 2018, according to the data provided by the National Institute of Statistics [31].

### Sampling and data collection

We collected the blood donors’ epidemiological data, including age, sex, rural or urban residency, occupational activity, recreational activities, animal husbandry (work or recreational) and exposure to tick bites. Sera were obtained prospectively and stored inmediately at −20 °C until serological assays were performed.

We estimated a total sample size of 440 participants, proportional to the population size of Castile-León according to the following criteria: an alpha value of 0.05 (95% confidence interval), a design effect of 1, assuming minimal household clustering, and a response rate of 90%. Finally, a representative group of 516 blood donors was selected for the serosurvey. The blood samples and epidemiological data were collected between May 2017 and May 2018 (Figure **).** The blood donors were healthy individuals between 18 and 65 years-old (they were not stratified by sex). The exclusion criteria were individuals with an established diagnosis of HIV infection, hepatitis B or C, syphilis, HTLV infection or malaria.

**Figure:**
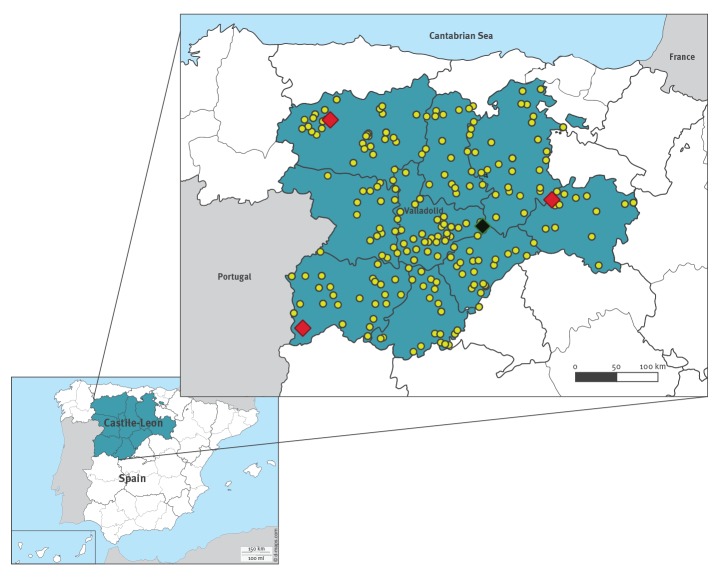
Geographical distribution of sample collection and positive results, Crimean-Congo haemorrhagic fever virus in blood donors, Castile-León, Spain, May–October 2017 and May–October 2018 (n = 516)

CCHFV: Crimean-Congo haemorrhagic fever virus.

Yellow circles: blood donors' places of residence; red diamonds: individuals with confirmed serology for IgG antibodies against CCHFV; black diamond: individual with a result concluded as indeterminate.

### Immunological assay

Antibodies against CCHFV were detected by a commercial immunoenzymatic kit (Vector Best, Novosibirsk, Russia). The cut-off was calculated as the mean of the adjusted optical density of the negative control serum plus 0.2. All samples with CCHFV-positive serology were tested in triplicate using the Vector Best test and the mean values of absorbance/cut-off were used for analysis. These positives samples were confirmed by an in-house ELISA described in Dowall SD et al. [32], an immunofluorescence assay (IFA) (Euroimmun, Lübeck, Germany)) using nucleoprotein (NP) and glycoprotein (GPC) antigens at 1:10 dilution. Likewise, we tested by all the above methods 50 randomly chosen samples that were initially negative from the 516 blood donors included in the study.

We considered samples as positively confirmed if they tested positive in at least two different tests. For IFA, we considered a result as indeterminate when the fluorescence result of the sample was not negative but less intense than that of the positive control incorporated in the test. 

### Data analysis

All data were statistically analysed using SPSS Statistics 23.0. For the qualitative variables, we calculated the proportions, and for the quantitative variables, we calculated the mean with standard deviation (SD) and median with interquartile range (IQR).

### Ethical statement

The procedures described here were carried out in accordance with the ethical standards described in the Helsinki Declaration revised in 2013. The protocol was approved by the Bioethics Committees: Comité Ético de Investigación Clínica Área de Salud Valladolid-Este, with the number/ID BIO-2017–65.

All participants signed an informed consent form. Each participant received this form, which contained the objectives, the procedures, the study characteristics and a question if they wanted to be a part of the study. We always maintained the confidentiality of the personal data of the blood donors. All samples were identified by a code.

## Results

A total of 516 blood donors participated in this study. The characteristics of the studied population are shown in Table 1.

**Table 1:** Characteristics of serosurvey participants, Crimean-Congo haemorrhagic fever virus in blood donors, Castile-León, Spain, May–October 2017 and May –October 2018 (n = 516)

Characteristics	n	%
Age (years), mean ± SD	46.3 ± 11.2	
Male sex	353	68.4
Professional activities at risk	109	21.1
Agriculture/shepherding activities	81	15.7
Slaughtering	6	1.2
Hunting	2	0.4
Veterinarians	1	0.2
Healthcare workers (nurses and laboratory technicians)	10	1.9
Rural population	448	86.8
Contact with animals	354	68.6
Animal husbandry practices	108	20.9
History of a tick bite	79	15.3

SD: standard deviation.

More than two thirds of the study participants were male (68.4%; 353/516), the mean age was 46.3 years (SD ± 11.2; range: 18–65 years). Most of the participants came from rural areas (86.8%; 448/516) and around two thirds had contact with animals (68.6%;354/516) and one fifth were involved in animal husbandry (20.9%; 108/516). One in five participants (21.1%; 109/516) were engaged in at-risk professional activities such as agriculture and shepherding activities, slaughtering, hunting, veterinary and healthcare work (mainly nursing staff and laboratory technicians). Seventy-nine participants (15.3%) were bitten by ticks in the period 3 days to 3 months before the date of sampling.

Positive serological results are presented in Table 2. There were six samples in which we found antibodies in at least one of the performed assays (0.96%) and three samples in which we found agreement in at least two of the performed assays (3/516), establishing a seroprevalence between 0.58% and 1.16%.

**Table 2:** Samples from blood donors positive for antibodies against Crimean-Congo haemorrhagic fever virus by ELISA and/or IFA, Castile-León, Spain, May–October 2017 and May –October 2018 (n = 6)

	ELISA IgG		IFA IgG		ELISA IgM		IFA IgM		Final result
	Vector Best^a^	In house^b^	GPC	NP	Vector Best^a^	In house^b^	GPC	NP	
1	10.01 (+)	3.4 (+)	−	−	0.50 (−)	0.3 (−)	−	−	+
2	10.01 (+)	0.1 (−)	−	−	0.01 (−)	Not done	Not done	Not done	−
3	10.01 (+)	6.4 (+)	Indeterminate	Indeterminate	0.01 (−)	Not done	Not done	Not done	+
4	2.59 (+)	0.2 (−)	−	−	0 (−)	Not done	Not done	Not done	−
5	0.90 (+)	0.5 (−)	+	Indeterminate	0.01 (−)	Not done	Not done	Not done	+
6	0.01 (−)	0.3 (−)	Indeterminate	+	0 (−)	Not done	Not done	Not done	Indeterminate

+: positive; −: negative; GPC: glycoprotein precursor construct; ELISA: enzyme-linked immunosorbent assay; IFA: immunofluorescence assay; NP: nucleoprotein.

ELISA results are expressed as a ratio sample absorbance/cut-off. IFA is expressed as a qualitative result obtained at 1:10 dilution.

^a^ This assay was performed in triplicate.

^b^ In*-*house ELISA performed at the National Centre of Microbiology.

The three individuals with positive IgG results were all men from rural areas in Castile-León (Figure). The ages of these individuals were 18, 44 and 60 years. None of them had a history of tick bites. Moreover, two of the three were involved in animal husbandry (either for professional or recreational reasons) and one performed outdoor activities putting them at risk of infection, such as trekking and mountain biking). It is important to mention that two other individuals were positive only by the VectoCrimea ELISA, and another one was positive only in the NP IFA with an indeterminate result in the GPC IFA (Table 2).

## Discussion

To our knowledge, this is the first study conducted in healthy blood donors in Spain, which attempts to describe the prevalence of this disease and evaluate the potential circulation of CCHFV. This study shows that CCHFV has infected humans in western Spain who did not recall having had any suggestive symptoms in the past. None of the individuals with IgG antibodies against CCHFV remembered being bitten by a tick, but all of them reported living in rural areas and being involved in animal husbandry. The absence of IgM antibodies in the three infected individuals suggests that there were no acute or recent infections in our sample population. However, our work had several limitations such as the small sample size. This could be involved in the fact that statistical significance was not reached when we looked for the possible risk factors implicated in the patients with positive serology. In addition, we did not perform all the serological tests available, for example seroneutralisation was not available in our laboratory at the time of the analysis, and we did not perform this test because it lacks sensitivity because of the weak neutralising antibody response to this virus [45,46].

The prevalence we found here (0.58–1.16%) is similar to that reported in the 1980s by Filipe et al. in southern Portugal [23]. It is also similar to the prevalence found in other European countries with a very low or null incidence, such as Hungary [35] or Greece [36]. Even though the seroprevalence in the general population in Greece is close to 3.5%, (with an unexpectedly high seroprevalence > 15% in individuals involved in animal husbandry for work), these values contrast with the low incidence of the disease on the Greek territory (only one symptomatic and fatal case has been reported since 2008) [36].

The classification of positive results in low-prevalence diseases is difficult, especially when only limited information is available about the performance characteristics of the analytical tests. Previous studies, such as the one performed by Vanhomwegen et al. in 2012 [37], showed that both enzyme immunoassay (EIA) and IFA used in this study have an acceptable sensitivity (80–85%) and an excellent specificity > 98% for both IgG and IgM. It is therefore unlikely that discrepancies between EIA and IFA results were due to the lack of specificity of these techniques. We decided to consider as positive only those samples that showed positive results in most tests performed (at least two of the four assays performed), in order to perform an accurate classification of the cases as positive, negative or indeterminate in the absence of a gold standard (a procedure used, for example, when studies are carried out to compare different diagnostic methods [38]). Therefore, we assume that the presented seroprevalence range is adequate, in accordance with the above performance allocation criteria. Protocols of the Advancing Transfusion and Cellular Therapies Worldwide (an association developing standards for transfusion medicine), have so far not established the need to detect CCHFV in blood or other blood-derived products [39]. The currently available data are insufficient to make an evidence-based recommendation for excluding blood donation product because we have not seen an individual with acute infection [40]. Although our study suggests that CCHFV is circulating in Castile-León, the data are, in the absence of IgM-positive results, not sufficient to re-evaluate the current statements about the screening of CCHF in blood donors. 

The geographical expansion to northern Europe of several vector-borne communicable diseases has increased in recent years. This phenomenon is associated with various factors including an increase in the global annual average temperature [41,42], human displacements, augmentation of population density in determined vulnerable zones [43,44], legal and illegal trade of animals and animal products [45] and patterns of land use for agriculture [46]. Recent cases associated with the African-III clade of CCHFV reported in Spain show that CCHF is not an exception [25]. The introduction of the virus in south-western Europe could be related to the transportation of infected larvae and nymphs of hard ticks by migratory birds from North Africa [24]. The ticks established their ecological environment in this zone where they became adults and fed on ungulates or human beings, maintaining an enzootic cycle [6]. Our results demonstrate the presence and circulation of the virus in western Spain, even with a low level of endemicity.

## Conclusion

This is the first serosurvey conducted in Spain that suggests a possible circulation of the virus in asymptomatic individuals. Our results suggests the need for more extensive studies, including a seroprevalence survey in wild or domestic animals and other serosurveys in people living in other areas of Spain in order to learn more about the epidemiology of CCHFV in Spain and to evaluate the relevance of control measures for clinical interventions in situations with a potential risk of transmission, such as blood or organ donation procedures.

## *Note

*This designation is without prejudice to positions on status, and is in line with United Nations Security Council Resolution 1244/99 and the International Court of Justice Opinion on the Kosovo declaration of independence.
